# The effects of stress on gut virome: Implications on infectious disease and systemic disorders

**DOI:** 10.1002/mbo3.1434

**Published:** 2024-09-23

**Authors:** Francesca Talarico, Bruno Tilocca, Rocco Spagnuolo, Ludovico Abenavoli, Francesco Luzza, Paola Roncada

**Affiliations:** ^1^ Department of Health Sciences University “Magna Graecia” Catanzaro Italy

**Keywords:** gut–brain axis, gut virome, host immune system, infectious diseases, stress, system disorders

## Abstract

The role of gut microbiota in health and disease is being thoroughly examined in various contexts, with a specific focus on the bacterial fraction due to its significant abundance. However, despite their lower abundance, viruses within the gut microbiota are gaining recognition for their crucial role in shaping the structure and function of the intestinal microbiota, with significant effects on the host as a whole, particularly the immune system. Similarly, environmental factors such as stress are key in modulating the host immune system, which in turn influences the composition of the gut virome and neurological functions through the bidirectional communication of the gut–brain axis. In this context, alterations in the host immune system due to stress and/or dysbiosis of the gut virome are critical factors in the development of both infectious and noninfectious diseases. The molecular mechanisms and correlation patterns between microbial species are not yet fully understood. This literature review seeks to explore the interconnected relationship between stress and the gut virome, with a focus on how this interaction is influenced by the host's immune system. We also discuss how disturbances in this finely balanced system can lead to the onset and/or progression of diseases.

## Graphical abstract

Stress conditions can weaken the host's immune system, affecting the entire gut–brain axis. Concurrently, disruptions in the composition of the gut microbiota are associated with exacerbations of diseases. This review explores the complex interplay between stress, gut virome, and the host immune system. It highlights how stress‐induced immune system alterations and gut virome dysbiosis contribute to both infectious and noninfectious diseases. The article further discusses the need for more research to fully understand the underlying molecular mechanisms and microbial correlations.

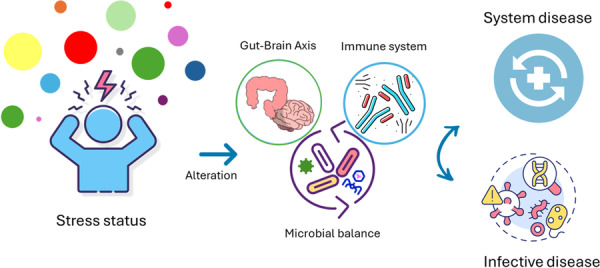

## 1 INTRODUCTION

The gut microbiota, which refers to the collection of microorganisms residing in the intestinal tract of animals and humans, represents a complex and dynamic ecosystem that significantly impacts the host's health throughout their life cycle (Kastl et al., 2020). The functions of the intestinal microbiota are diverse: it ensures the proper functioning of the intestine, synthesizes vitamins (primarily B group vitamins), and neutralizes toxins, xenobiotics, and carcinogenic compounds (Góralczyk‐Bińkowska et al., 2022). Moreover, these microorganisms participate in food digestion by producing short‐chain fatty acids (SCFA) from the fermentation of dietary fibers (Martin‐Gallausiaux et al., 2021). The intestinal microbial community also forms a protective barrier against pathogens (Tilg et al., 2022) and performs immunomodulatory functions, coordinating the immune response to physical and/or biological insults and promoting the maturation and homeostasis of the child's immune system (Cristofori et al., 2021). These processes result from the intricate network of interactions between different components of the microbial community and their host, as well as the effects of environmental and host‐specific variables on the microbial community (Kuziel & Rakoff‐Nahoum, 2022). Given this, numerous studies are currently being conducted to elucidate the mechanisms and dynamics of the intestinal microbiota in various physiological and pathological processes, including the study of its structural and functional shaping to support health in animals and humans (Kuziel & Rakoff‐Nahoum, 2022).

Most studies have primarily focused on characterizing the bacterial component of the microbiota, with only marginal attention given to other microbial components such as viruses. Despite their lower abundance, viruses play a crucial role in maintaining the homeostasis of the superorganism, through both direct and indirect effects on the overall microbiota composition and activity (Lecuit & Eloit, 2017). Therefore, there is a pressing need to expand current research to include the intestinal virome, which is still poorly characterized (Matijašić et al., 2020). The intestinal microbiota, including the viral fraction, is influenced by several variables, such as dietary habits (Adak & Khan, 2019), metabolic disorders (Abenavoli et al., 2022; Abenavoli, Scarlata, et al., 2023; Abenavoli, Scarpellini, et al., 2023; Lloyd‐Price et al., 2019), and changing environmental conditions (Hsu & Schnabl, 2023). These factors shape communication axes with different organs or body districts (Ahlawat et al., 2020) such as the gut–lung (Budden et al., 2016; Enaud et al., 2020; Zhang et al., 2020), gut–skin (Pessemier et al., 2021; Thye et al., 2022), and gut–brain axes (Manos, 2022). There is solid evidence of the functional connection between the intestinal microbiota and the host's brain. Investigations into the “gut–brain axis” primarily focus on pathological alterations of the central nervous system. Numerous studies confirm that the intestinal microbiota is involved in neurological and mental disorders (i.e., Alzheimer's disease, dementia, anxiety, depressive disorder, Parkinson's disease, schizophrenia) (Generoso et al., 2021). However, only a few studies are available on the role that diverse stress conditions have on the gut microbiota and the viral fraction in particular (Kraimi et al., 2022; Kumar et al., 2023; Tan, 2023; Xiong et al., 2023). Deepening this aspect could yield further knowledge on the gut microbiota, providing additional practical implications. Elucidating the mechanisms by which stress affects gut‐associated microorganisms (particularly viruses) adds impactful pieces of knowledge. These, in turn, enable tailoring the bidirectional communication network occurring between the brain and the gut microbiota, as mediated by the host immune system, thus opening for “indirect” control of the linked downstream conditions.

This review aims to provide an overview of the current studies characterizing the effect of various stress conditions on the gut microbiota, elucidating the physiological implications of the viral fraction, and how its alteration may trigger the onset of both infective and noninfective conditions.

## 2 GUT MICROBIOTA COMPOSITION

The adult human intestine is home to approximately one trillion (10^18^) microbial specimens, including bacteria, fungi, archaea, and viruses. Bacteria are the primary colonizers of the gut, with up to 1000 different species (Gomaa, 2020). However, only five phyla predominantly compose the human microbiota: Firmicutes, Bacteroidetes, Actinobacteria, Proteobacteria, and Fusobacteria (Weiss & Hennet, 2017a). The proportions of each taxa vary dramatically between individuals and even within an individual throughout their lives, making each person's microbiome unique (Davenport et al., 2017). However, the concept of a “core microbiota” (i.e., the most abundant and conserved fraction of the microbial community, identified using a genomic approach) (Madhu et al., 2023) is likely, and some research groups may classify the adult's overall taxonomic profile into a limited number of well‐balanced host–microbiota symbiotic states, known as enterotypes (Divella et al., 2021).

Fungi, which mainly belong to three phyla: Ascomycota, Basidiomycota, and Zygomycota, cover a relatively small percentage of the human microbiota. They play a crucial role in modulating intestinal homeostasis and are widely used as probiotics. Like bacteria, reduced fungal diversity and dysbiosis are associated with pathological states, such as visceral hypersensitivity, a key feature of inflammatory bowel disease (IBS) (Gomaa, 2020).

The Archaea domain includes a wide variety of microorganisms that share properties with both bacteria and eukaryotes (Gaci et al., 2014). Methanogenic archaea are the most common archaeal specimens in the human and animal gastrointestinal tract (Saengkerdsub & Ricke, 2014). These strict anaerobes are found in various sources, including the environment (De Vos et al., 2022), and their physiology and ecology are extensively studied due to their intrinsic ability to produce methane, which has significant implications for environmental pollution (Conrad, 2009). However, the archaea responsible for methane production are largely overlooked in human microbiome studies due to their nonbacterial biology, leading to detection issues (Hoegenauer et al., 2022).

The gut virome encompasses the population of eukaryotic viruses, bacteriophages, endogenous retroviruses, and archaeal viruses (Mukhopadhya et al., 2019). Although largely unexplored, the viral fraction is known to shape the gut bacteriome. Bacteriophages actively modulate the bacterial population by directly infecting them. Additionally, enteric viruses are the main vehicle for horizontal gene transfer, thus indirectly influencing bacterial evolution, diversity, and metabolism (Lee & Baldridge, 2019; Minot et al., 2013; Mukhopadhya et al., 2019). Alongside this, the viral fraction regulates the host immune system in a manner complementary to the bacterial fraction. Specifically, eukaryotic viruses can swiftly move through the layers of the epithelial cells, influencing the development of immune cells and lymphocytes, which constitute the first line of defense against intestinal pathogen colonization (Lee & Baldridge, 2019). This, in turn, regulates the release of immunomodulatory compounds dependent on the bacterial metabolites.

### 2.1 Gut virome

The virome, which refers to the collection of viruses that make up the intestinal microbiota, represents 1% (or less) of the total metagenomic reads, according to metagenomic studies on human fecal samples. It is estimated that every gram of feces contains 10^8^–10^9^ virus‐like particles (VLP) (Iliev & Cadwell, 2021). The human intestinal virome includes both eukaryotic viruses, which replicate by infecting human cells, and bacteriophages, which replicate by infecting bacteria (Lecuit & Eloit, 2017). The eukaryotic virome primarily consists of the families Adenoviridae, Anelloviridae, Astroviridae, Parvoviridae, Picornaviridae, and Picobirnaviridae (Matijašić et al., 2020). Eukaryotic viruses are acquired progressively with age, unlike bacteriophages, which are highly present in the early stages of life and then decrease in their relative abundance over time (Lim et al., 2015). Bacteriophages constitute the most substantial component of the intestinal virome, and their presence is strongly influenced by the presence of their bacterial hosts (Lecuit & Eloit, 2017). Despite this, the most abundant are the DNA viruses, particularly Caudovirales (double‐strand DNA viruses) and Microviridae (single‐strand DNA viruses) (Cao et al., 2022). Bacteriophages are divided into lytic and lysogenic based on the type of interaction established with the bacterial host, although some can perform both lytic and lysogenic cycles (e.g., *Escherichia coli* lambda phage). During the lytic cycle, a bacteriophage infects a live bacterial target cell, replicates inside it, kills the bacterium through lysis, and releases hundreds or thousands of new bacteriophages, potentially playing a role in the vehiculation of genic traits (Żbikowska et al., 2020). For example, an investigation conducted on the growth cycle of the lytic bacteriophage k3w7, a ds‐DNA virus belonging to the myoviridae family, potentially useful in therapies against *Klebsiella pneumoniae* infections, has been shown to produce carbapenemase (Baqer et al., 2023). Also, results showed that this virus has a short latency period (approximately 20 min) and a burst size of approximately 220 plaque‐forming units per infected cell (Baqer et al., 2023). This strategy enables a swift modulation of the overall microbiota composition, by acting either directly or indirectly on the most abundant microbial fraction (i.e., bacteria), besides conditioning the biology of both the microbiota and the host (Hsu et al., 2019). Quantitative modulation of the microbiota composition has been attributed to bacteriophages at the expense of the low‐ and high‐abundant bacterial specimens in a strictly specific manner (Reyes et al., 2013). In turn, the altered quantitative composition is mirrored in third, nontargeted, microbial specimens as the consequence of the changing biotic and abiotic milieu (Hibbing et al., 2009; Hsu et al., 2019; Schmidt et al., 2018).

In contrast, the lysogenic cycle leads to the integration of the viral genome, in a prophage form, into the bacterial chromosome (Żbikowska et al., 2020). It has been observed that *E. coli* prophages encode genes for resistance to antibiotics and other environmental stresses (Minot et al., 2011), underlying the interdependencies among the microorganisms within the ecological niche. Additionally, bacteriophage members can be classified as monovalent, infecting only one bacterial species, and polyvalent phages, capable of infecting two or more bacterial species (Żbikowska et al., 2020). Nevertheless, this classification is somewhat arbitrary since it is based on in vitro tests relying on a limited number of bacterial isolates, thus limiting the definition of the bacteriophage's tropism itself, which is likely to change in the context of the human gut (Chung et al., 2023; Kim et al., 2021).

## 3 ALTERATION OF THE GUT MICROBIOTA

Under normal conditions, the microbial community members, both pathogenic and nonpathogenic, exist in a state of structural and functional balance, known as eubiosis. This state is characterized by nonpathogenic microorganisms controlling pathobiont microorganisms, keeping their pathogenic state inactive and including them in the intricate network of interactions that enable the homeostatic status of the superorganism (Iebba et al., 2016). However, this is a dynamic equilibrium where host‐related and environmental variables guide the continuous remodeling of the microbiota in terms of both structure and function (Mukhopadhya et al., 2019). The data available from literature surveys are quite heterogeneous, with some studies supporting a strong modulatory effect of the diet (Minot et al., 2011, 2013), while others attribute minor importance to the diet as a challenging factor (Reyes et al., 2015). Interestingly, compared to the bacterial counterpart, the virome appears more stable over time at the individual level (Reyes et al., 2015; Norman et al., 2015). Other environmental factors, such as the administration of antibiotics, are involved in significant changes in the virome composition. These predominantly act on the bacteriophage population by reducing the load of available hosts to the bacterial specimens carrying antimicrobial‐resistant traits (Modi et al., 2013).

Drastic environmental changes or sudden alterations of the host biochemistry lead to the disruption of the balance, known as dysbiosis, and vice versa (Greer et al., 2016). The mechanisms underlying such intestinal dysbiosis are often unclear due to the multitude of variables influencing the complex microbial composition simultaneously. Indeed, it is a multifactorial condition resulting from the combination of alterations in the intestinal environment and stress factors, mediating destabilizing cascades of events leading to dysbiosis (Weiss & Hennet, 2017). In this scenario, oxidative stress, bacteriophage induction, and the secretion of bacterial toxins can trigger rapid changes among intestinal microbial groups, causing a state of dysbiosis (Weiss & Hennet, 2017).

### 3.1 Factors of virome dysbiosis

A recent study by Nishijima et al. identified as many as 97 factors that influence the virome structure. These factors are intrinsic (age, sex) or extrinsic (lifestyle, diet), most of which concern both bacteriophages and their bacterial hosts. These represent a basis for understanding the symbiotic relationships occurring among the bacterial communities and their viruses, supporting the medical and industrial applications of indigenous viruses (Nishijima et al., 2022). Mounting evidence suggests that the human gut virome displays distinct, age‐dependent patterns of diversity, in response to an array of factors including immune status fluctuations throughout life stages (infant, child, adult, elderly). The highest overall viral richness is observed in infants and adults: there are significant decreases between infants and children or adults and elderly individuals, but there are significant increases between children and adults. These overall trends, however, did not apply evenly across virus types. For example, eukaryotic virus richness (mostly human Anelloviruses) is high in infancy, presumably driven by an underdeveloped immune system, and then decreases into childhood and remains constant and low through the rest of life. The bacteriophage family Siphoviridae (the most abundant in the human microbiota) mirrors the overall trend, but on the contrary, Microviridae reach a modest peak in infancy, decrease in childhood, and then slowly increase across the rest of the lifespan (Gregory et al., 2020).

Experiments on subjects who followed a different diet have shown that the intestinal virome changes significantly based on the type of diet. The pre‐existing proportions in the virome were altered, while the subjects who followed the same diet had a more similar composition of the virome (but not identical because of the interpersonal variation). Specifically, dietary intervention suggests a trend toward enrichment in Siphoviridae and depletion of Myoviridae (Minot et al., 2011), although tailored changes are expected to happen depending on the change and duration of the dietary intervention. Interestingly, a study analyzing the fecal virome of 930 healthy adult subjects from two regions in China (Hong Kong and Yunnan), spanning six ethnicities (Han, Zang, Miao, Bai, Dai, and Hani), and including urban and rural residents for each ethnicity observed that gut‐DNA virome differed significantly with geography‐ and ethnicity‐specific compositional differences. Similarly, bacterial community composition was significantly affected, although overall human gut virome was more heterogeneous than the gut bacteriome at the population level (Zuo et al., 2019, 2020).

Gut virome differences have been linked to the presence of other pathological conditions. For example, in stroke patients, an alteration in the structures and composition of the microbiome was observed, which is probably due to changes in the virome. In fact, in these subjects, there was an increase in the relative abundance of Bacteroides phage B40_8 and Cronobacter phage CS01. Furthermore, in patients' virome, the relative abundance of the Streptococcus phages was positively correlated with that of their hosts, while in the healthy volunteers' virome, the relative abundance of the Faecalibacterium phage, Bilophila phage, and Roseburia phage was positively correlated with that of their hosts. These underline the connection existing between the composition of the bacteriophage and the bacterial fraction (Wang et al., 2021; Zhang et al., 2024).

## 4 GUT VIROME IN THE GUT–BRAIN AXIS

The gut–brain axis refers to a “bidirectional communication” in which the intestinal microbial community influences the neurophysiological activities of the central nervous system (CNS), while specific conditions of the CNS are reflected in the structural and functional organization of the intestinal microbiota (Mayer et al., 2022).

**Figure 1:**
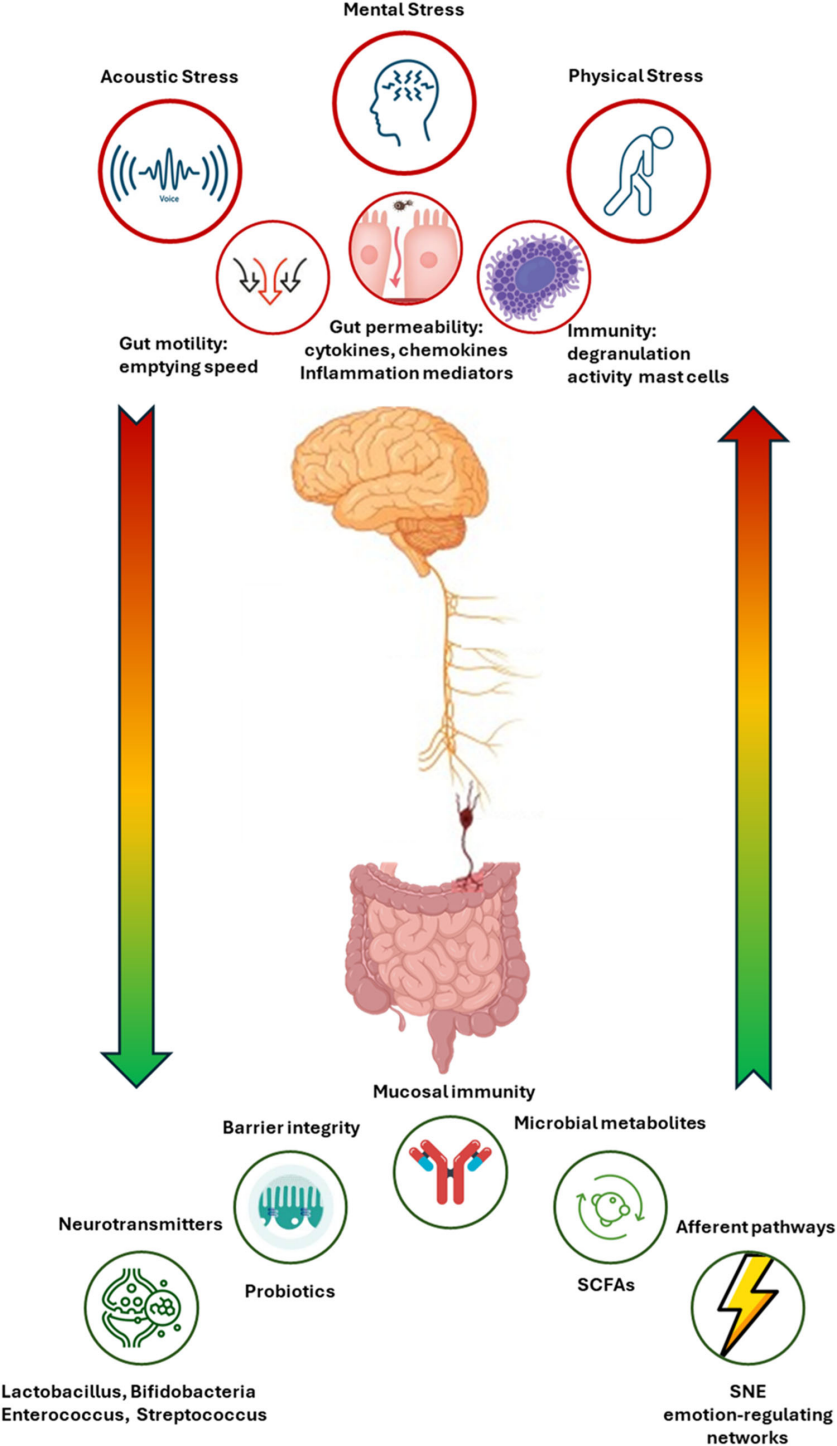
The gut–brain axis. The figure summarizes the major routes undertaken by the brain and the microbial fraction harboring the gut microbiota in the bidirectional crosstalk. SCFAs, short‐chain fatty acids; SNE, enteric nervous system.

This bidirectional communication is mediated by immune, neuroendocrine, and neural pathways (Figure 1). Specifically, the gut microbiota influences the brain through several mechanisms:

1. The production, expression, and turnover of neurotransmitters, including acetylcholine, gamma‐aminobutyric acid (GABA) (Strandwitz, 2018), and serotonin produced by bacteria belonging to the genus Lactobacillus, Bifidobacteria, Enterococcus, and Streptococcus. These neurotransmitters are involved in communication patterns within the intestinal microflora and also have systemic and peripheral effects that influence brain function (Góralczyk‐Bińkowska et al., 2022).
2. Maintenance of intestinal barrier integrity and tight junctions. Species‐specific probiotics have been shown to restore tight junction integrity and protect the intestinal barrier (Wilkins & Sequoia, 2017).
3. Modulation of afferent sensory pathways of the enteric nervous system. The main pathway is the afferent branch of the vagus nerve, which connects the gut to the nucleus of the solitary tract and to the higher mammalian brain networks that regulate emotions (Breit et al., 2018).
4. Production of microbial metabolites. Short‐chain fatty acids (SCFA) are among the main products of bacterial metabolism (Rooks & Garrett, 2016).
5. Mucosal immune regulation. Altered microbiota activates innate mucosal immune responses, increasing epithelial permeability, activating nociceptive sensory pathways that induce visceral pain, and disrupting the enteric nervous system (Carabotti et al., 2015).

The brain influences the microbiota through several mechanisms:

1. Alteration of motility. External factors such as acoustic stress affect motility by slowing the recovery of the migratory motor complex pattern and inducing a transient decrease in the rate at which gastric emptying occurs (Carabotti et al., 2015).
2. Alteration of intestinal permeability. Stress or psychological distress impair barrier integrity leading to increased permeability and an enhanced leakage of bacteria and endotoxins into the system circulation, contributing to systemic inflammation and various pathological conditions.
3. Alteration of immune function. Mast cells are influenced by the sympathetic branch of the autonomic nervous system, which modulates their charge, degranulation, and activity; in cases of stress‐related muscle dysfunction, this pathway results in an imbalance in the release of tryptase and histamine. Some mast cell products may increase epithelial permeability to bacteria, facilitating their access to immune cells in the lamina propria (Carabotti et al., 2015).

## 5 STRESS AS A DYSBIOSIS FACTOR

Stress is a psychophysical response to events of an emotional, cognitive, or social nature that are perceived by the individual as excessive. Depending on the duration of the stressful event, it can be classified as acute or short‐term stress if the stimulus occurs only once and has a limited duration, and chronic stress if the source of stress persists over time. In acute stress, the body activates stress response mechanisms, which play a crucial role in cellular survival, intestinal barrier integrity, and immune system modulation. Long‐term stress, such as the chronic pain of uncontrolled fibromyalgia, causes immune dysregulation by disrupting the proliferation and maturation of immune cells and causing an imbalance in type 1 and type 2 cytokines (Freidin et al., 2021; Gasmi et al., 2022; Piras et al., 2022).

The mechanisms by which stress induces dysbiosis, and susceptibility to infectious diseases, vary depending on the source of stress. Generally, the occurrence of stress factors, as well as conditions of dysbiosis, leads to the activation of the hypothalamic–pituitary–adrenal (HPA) axis, inducing an increase in the secretion of corticotropin‐releasing hormone (CRH) from the hypothalamus. This triggers the release of adrenocorticotropic hormone (ACTH) from the anterior pituitary gland, which then stimulates the adrenal gland to produce and secrete the stress hormone cortisol. Cortisol is responsible for modulating immune functions and the intestinal barrier, with repercussions on the composition and function of the intestinal microbiota (Góralczyk‐Bińkowska et al., 2022). A recent study by Ritz and colleagues demonstrated that chronic social stress increases bacteriome and virome beta diversity compared to control fecal samples. The authors emphasized the role of stress in reducing the efficiency of the host immune system. Stress effects mediated by the gut virome are reflected as altered immune cell circulation, cytokine array production, and impaired transcriptional profile at the amygdala level. This results in a more permissive immune system, enabling the colonization and growth of pathogenic bacteria and even the shift in ecological behavior of the pathobionts (Ritz et al., 2024). Conversely, other studies highlighted that viral infections are linked with elevated levels of proinflammatory cytokines associated with schizophrenia (Comer et al., 2020; Kępińska et al., 2020), supporting the strict linkage between the virome and brain health status through the immune system (Pargin et al., 2023). Also, exposure to chronic stress is a significant risk factor in the pathogenesis of various gastrointestinal diseases, including functional dyspepsia, inflammatory bowel disease, gastroesophageal reflux disease, peptic ulcer, and irritable bowel syndrome (Konturek et al., 2011).

### 5.1 Stress‐induced susceptibility to infectious diseases through virome alteration

Both stress and the virome are considered significant factors in hindering the functions and efficiency of the immune system. In this scenario, two independent factors have converging outcomes, that is, host immune suppression, enabling the growth of pathogens and/or pathobionts, and flare‐ups of infections.

The impact of stress on the gut virome is evaluated by considering the duration of the stress period. There is a notable difference in the effects of acute and chronic stress. Acute stressors (e.g., lasting minutes) can cause downregulation of specific immunity and upregulation of natural immunity. For example, mental stress due to exams tends to suppress cellular immunity and preserve humoral immunity. In contrast, chronic stressors are associated with decreased functionality of both cellular and humoral immunity. Depending on the type of stressful situation, the sequence of events affecting the immune system (trauma or loss) may vary. Nevertheless, diverse responses to the same stressor (and stressor entity) have been observed depending on subjective variables (e.g., age). This, in turn, is mirrored as variable immunological impairment (Segerstrom & Miller, 2004). A study performed on rats observed that a brief acute restraint stress led to an increased number of memory T‐cells before immunization and helper T effector cells after immunization, suggesting short‐term stress as an important immune booster and an effective strategy for the control of infective agents. This also appears to influence a more robust immune response following repeated stimulation with the same antigen even after months (Vitlic et al., 2014). Chronic stress, on the other hand, is generally recognized as immunosuppressive (Sorrells & Sapolsky, 2007), negatively influencing pathological outcomes of both infectious and neoplastic diseases. Also, allergic and autoimmune disease outcomes are affected by prolonged stress. Although apparently in contrast, one possible explanation suggests that chronic stress alters cytokine expression patterns, causing the suppression of Th1 cytokines (such as IFN‐γ), involved in defense against intracellular infections and some neoplastic diseases, and the activation of Th2 cytokines (such as IL‐10), involved in the onset and progression of allergies and various autoimmune diseases (Vitlic et al., 2014). Additionally, it has been demonstrated that acute stress increases the lymphocyte count in the blood, while chronic stress causes an overall decrease in the number of circulating lymphocytes, but there is an increase in the number of immature T lymphocytes. This suggests one of the potential mechanisms through which the immunosuppression associated with chronic stress can influence and exacerbate autoimmune diseases (Moroda et al., 1997).

In summary, acute stress factors, except for natural killer (NK) cells and superoxide production by neutrophils, enhance the immune system, particularly its innate component that can act rapidly. In any case, the greatest number of chronic stress factors are associated with general immunosuppression which affects both components of the immune system.

Evaluating antibody titers against latent viruses is an effective method for analyzing the consequences of psychological stress on the immune system (Vitlic et al., 2014). Stress resulting from marriage discordances negatively affects endocrine responses and immunological activation. An increase in antibody titers against the Epstein–Barr virus (EBV) and the blastogenic response to mitogens of T cells was observed in both men and women (Kiecolt‐Glaser et al., 1997). Another study analyzed a group of caregivers and higher antibody titers against cytomegalovirus (CMV) were observed, indicating poorer control of the latent virus due to psychological stress events (Pariante et al., 1997).

It is often thought that eukaryotic viruses are responsible exclusively for asymptomatic infections, but active viral replication can influence the immune system, with positive or negative outcomes, depending on the circumstances (Iliev & Cadwell, 2021). Moreover, bacteriophages can transfer, through their nucleic acid, genes for antibiotic resistance or genes that code for the immune system escaping, thus increasing enteric bacterial pathogenicity (Colomer‐Lluch et al., 2011). For example, phages sakϕC and sak42D (*Staphylococcus aureus*) encode staphylokinase (sak) that neutralizes antimicrobial peptides thus eluding the immune system and causing host tissue death (Nguyen & Vogel, 2016; Van Wamel et al., 2006). Phages can also encode inhibitors of complement (SCIN), proteins (CHIP), superantigens, and other substances that are responsible for the lethal outcome of *S. aureus* infection (De Jong et al., 2018). Also, emerging *S. aureus* strains can become resistant to ampicillin thanks to β‐lactamase genes, blaTEM and blaCTX‐M9, that can be transferred by *S. aureus* phage. *Salmonella typhimurium* strains appear to be multiantibiotic resistant acknowledging the transfer of resistance genes by temperate phages (Colomer‐Lluch et al., 2011).

A study on the role of virome in bacterial adaptation, after antibiotic‐induced stress in animal models revealed that phage genes undergo robust enrichment following antibiotic treatment. Such gene enrichment could promote the production of resistance genes against the administered drug in phages and their subsequent transfer to the bacterial community, thereby acting as a reservoir for resistant strains (Spencer et al., 2022). For example, research about patients undergoing antibiotics therapy for Helicobacter pylori demonstrates that following Helicobacter pylori eradication, gut virome diversity decreased (especially after multiple treatment cycles), community diversity increased, and there was a greater proportion of core viruses. The duration of these effects is 6 months, while phage–bacteria interactions are enhanced for up to 6 weeks (Wang et al., 2023) (Table 1).

**Table 1:** Effect of phages and stress on the immune system.

Condition	Consequences	References
Phage infection		
SakϕC and sak42D (*Staphylococcus aureus* phage)	Encode for the immune regulator staphylokinase (*sak*) which neutralizes antimicrobial peptides	Nguyen and Vogel (2016); Van Wamel et al. (2006)
*S. aureus* phage	Transfer β‐lactamase genes, *blaTEM* and *blaCTX‐M9,* conferring ampicillin resistance to new *S. aureus* strains	Colomer‐Lluch et al. (2011)
Temperate phages	Transfer multiantibiotic‐resistant genes, conferring resistance to *Salmonella typhimurium* strains	Colomer‐Lluch et al. (2011)
Stress		
Acute stress	Enhance the immune system by increasing the number of memory T‐cells before immunization and T‐helper effector cells after immunization.	Moroda et al. (1997); Vitlic et al. (2014)
	Increases the lymphocyte count in the blood	
Chronic stress	Immunosuppression alters cytokine expression patterns, causing the suppression of Th1 cytokines and the activation of Th2 cytokines.	Moroda et al. (1997); Vitlic et al. (2014)
	Decreased the lymphocyte count in the blood, but increased the number of immature T lymphocytes	

Nevertheless, information regarding virome and the immune system is still insufficient. A possible explanation relies on the fact that the immune system normally suppresses these viruses to an abundance below the limit of detection of the most sensitive technologies, such as metagenomics (Ouabbou et al., 2020). The effects of intestinal viral infections are often mediated by interferons (IFNs), particularly type I, such as IFNA and IFNB, and type III, such as IFNLs (Sun et al., 2015). On the other hand, in case of infection by a virus that establishes symbiotic relationships, the IFN response may be necessary.

### 5.2 Implications of stress‐altered virome on systemic disorders

Both mental and psychological stress are acknowledged to influence the host immune system and thus, the balance of the host‐virome. It is straightforward to assume a cascade effect on the emergence and progression of systemic disorders, in addition to those of infectious origin.

Studies in subjects with type 1 diabetes provide the strongest evidence that intestinal viruses can cause chronic immune disease in humans (Notkins et al., 1979). Autoantibodies against insulin have been associated with chronic enterovirus B infection and, in a smaller number of cases, mastadenovirus C infection. Small single‐stranded DNA viruses belonging to the Circoviridae family have also been associated with the development of diabetes‐associated serum antibodies in the absence of early infection. Infection with group B Coxsackievirus reduces disease in younger nonobese diabetic (NOD) mice but accelerates it in older mice at a time when insulin values are already altered. Rotaviruses accelerate the autoimmune reaction by stimulating the production of type I INF by the pancreas, where it arrives through the lymph nodes; IFNs, in turn, activate self‐reactive lymphocytes. Murine norovirus (MNV) infection, on the other hand, protects against the development of diabetes in NOD mice (Iliev & Cadwell, 2021).

Altered bacteriophage composition is associated with pathological inflammatory conditions affecting the intestine (Gogokhia et al., 2019). Specifically, clonal expansion of Caudovirales bacteriophages has been observed in patients with Crohn's disease (Norman et al., 2015). It has also been noted that, in murine models, viral infections induce characteristics associated with Crohn's disease and celiac disease. Particularly, they induce defects in Paneth cells and the synthesis of gluten‐reactive lymphocytes (Iliev & Cadwell, 2021). The increase in bacteriophage levels (mainly those with tropism toward *E. coli*, Lactobacillus, and Bacteroides) in germ‐free mice can alter the mucosal immune status and exacerbate colitis by inducing the production of IFN‐γ through TLR9 (Gogokhia et al., 2019). Furthermore, the Spounaviridae phages and those targeting Streptococcus and Alistipes have been proposed as informative markers for murine colitis analogous to IBD (Duerkop et al., 2018). Interestingly, increased levels of bacterial hosts were not observed, as opposed to previous studies on this topic, suggesting that phage abundance is not always correlated with bacterial abundance and that inflammation might trigger phage replication. Furthermore, a comparison of the metagenomes of experimental and human models of intestinal bacteriophages showed good correspondence, suggesting how the murine colitis model could be employed in translational medicine studies for the human model (Matijašić et al., 2020).

Inflammatory bowel diseases (IBD), encompassing two major forms: ulcerative colitis (UC) and Crohn's disease (CD), are closely associated with alterations in the gut–brain axis and stress. Previous studies, using patient‐reported outcomes (PROs) (Liuzza et al., 2021; Spagnuolo, Basile, et al., 2022), have highlighted how anxiety, depression, fatigue, and satisfaction in social roles may be compromised in these diseases both during flare‐ups and during clinical remission phases (Iaquinta et al., 2022; Mancina et al., 2020; Spagnuolo, Iaquinta, et al., 2022). Also, these factors worsen the natural history of the disease in terms of surgical recourse and hospitalizations (Fairbrass et al., 2022). In addition, the evolution of IBD is strictly linked to the effects that stress has on the gastrointestinal microbiota (Konturek et al., 2011). Increased microbial load in the colon tissue, excessive cytokine release, and partially attenuated immune reactivity due to stress are among the leading factors with a negative impact on IBD. Depression is associated with elevated levels of C‐reactive protein and tumor necrosis factor‐alpha (TNF‐α) in patients with IBD (Brzozowski et al., 2016). An increasing amount of data confirms that disturbances in the eukaryotic virome are linked to IBD pathogenesis: a study analyzing colon samples from IBD patients and healthy controls has shown that IBD is characterized by increased levels of the Herpesviridae family, as well as increased expression of relevant endogenous viral sequences (Wang et al., 2015). Studies on mice carrying mutations in IL‐10 or Atg16L1 demonstrate that eukaryotic viruses can encourage the emergence of IBD by interacting with risk genes (Basic et al., 2014). The enteric virome is also abnormal in Crohn's disease and ulcerative colitis patients (Norman et al., 2015). Patients with UC exhibit a higher abundance of Pneumoviridae compared to the control group, while the opposite is observed for the Anelloviridae family (Zuo et al., 2019). There is elevated abundance of Hepadnaviridae and Hepeviridae in ulcerative colitis patients compared to controls, while diet‐related viral families Polydnaviridae and Tymoviridae are less enriched. A similar trend has been observed for Virgaviridae in CD patients (Ungaro et al., 2019). Intestinal inflammation caused by eukaryotic viruses has also been studied in animal models, with a primary focus on Norovirus (Matijašić et al., 2020).

Based on current achievements, we can hypothesize that specific variations in the viral component of the microbiota could lead to IBD and that stress, in turn, could have a negative impact, causing disease flare‐ups or exacerbation of symptoms. On the other hand, stressful conditions may induce inflammatory states that could lead to IBD, potentially affecting the viral component of the microbiota. The table below summarizes the main alterations of the virome associated with major system and gastrointestinal diseases (Table 2).

**Table 2:** Virome alterations on the major human systemic disorders.

Disease	Virome alterations	References
Crohn disease	Expansion of *Caudovirales* phages and norovirus increases the probability of disease and increases the frequency of *Anellovirus*.	Axelrad et al. (2018); Axelrad et al. (2019); Clooney et al. (2019); Norman et al. (2015); Nyström et al. (2013); Ungaro et al. (2019)
Ulcerative colitis	Expansion of *Caudovirales* phages and increased frequency of *Anellovirus.*	Clooney et al. (2019); Norman et al. (2015); Tokarz et al. (2019); Ungaro et al. (2019); Zuo et al. (2019)
Celiac disease	High titers of antireovirus antibody and association of rotavirus infection with the development of the disease.	Bouziat et al. (2017); Stene et al. (2006)
Inflammatory bowel disease	Increased *Anelloviruses, Caudovirales*, temperate bacteriophages	Cao et al. (2022)
	Decreased *Microviridae*	
Obesity	Increased frequency of *Phycodnaviridae*, *Mimiviridae* (eukaryotic virus), *Microviridae*, and *Caudovirales* (bacteriophages)	Cao et al. (2022)
	Decreased frequency of *Siphoviridae* phages (in mice)	
Type 1 diabetes	Increased genomic sequences of eukaryotic viruses belonging to families of *Circoviridae* and *Picornaviridae* (including *Enterovirus, Kobuvirus,* and *Parechovirus*)	Cao et al. (2022)
	Decreased gut phageome richness, in particular, α‐diversity of *Podoviridae* and richness of *Myoviridae*	
Type 2 diabetes	Increased frequency of *Siphoviridae, Podoviridae, Myoviridae, Caudovirales, Escherichia phage, Geobacillus phage, and Lactobacillus phage*	Cao et al. (2022)
Clostridium difficile infection	Increased abundance of *Caudovirales* and *Anelloviridae*	Cao et al. (2022)
	Decreased diversity and abundance of *Microviridae*	
Colorectal cancer	Eukaryotic viruses: increased *Epstein‐Barr virus, human Papillomavirus, human Polyomavirus, Cytogamelovirus*	Cao et al. (2022)
	Bacteriophages: increased richness and diversity of intestinal phageome, increased abundance of temperate phages, increased *Inovirus* and *Tunalikevirus*, decreased of *Enterobacteria phages* and *crAssphages*	
Adrenoleukodystrophy	Increased *Parvoviridae, Herpesviridae, Enterobacteria phages, Escherichia phages*, and *Enterococcus phages*	Cao et al. (2022)

Taken together, the above observation suggests a potential implication of modulating the gut virome to address or at least ameliorate, these system disorders. Interestingly, a recent study demonstrated fecal virome transplantation (FVT) as a suitable method for restoring the gut microbiota of mice exposed to social chronic stress, with a consequent improvement of the clinical manifestation (Ritz et al., 2024). Similarly, FVT has proven successful in the remission of recurrent Clostridium difficile infections (Kao et al., 2019). Also, virome transfer from lean‐to‐obese mice restored the weight gain effect of the obese phenotype (Rasmussen et al., 2020). Similarly, targeted approaches such as phage therapy resulted in the selective reduction of Fusobacterium nucleatum, a bacterial specimen commonly associated with colorectal cancer conditions (Zheng & Dong, 2019); besides scoring success in reducing rates of antimicrobial resistance specimens, whose diffusion is currently posing serious threats to a broad spectrum of pathological conditions of both infectious and noninfectious origin (Duan et al., 2019; Heuler et al., 2021).

However, viral infections alone are not sufficient to induce a confirmed multifactorial disease. Viruses can, in combination with additional environmental factors and commensal bacteria, determine the phenotype of hosts carrying inflammatory diseases (Cadwell et al., 2010). The need for additional factors could explain the reduced frequency of association of common viruses with confirmed diseases (1% or less of the population). Moreover, studies in murine models of Crohn's and Celiac disease describe that the effects of viruses are strain‐specific, complicating the linkage of viral profiles with various pathological conditions (Ottman et al., 2012).

## 6 DISCUSSION

The microbial community residing in the intestinal environment is involved in dynamic cross‐relation networks with other parts of the superorganism, including the brain. The reshaping of the structure and functions of the gut microbiota is a key strategy to promptly respond to the physicochemical changes triggered by a myriad of variables, both host‐dependent and host‐independent (Hou et al., 2022). The investigation of the viral fraction holds the potential to provide impactful explanations on the role of various kinds of stress in the modulation of the gut microbiota associated with the occurrence of pathological conditions, both infectious and noninfectious.

Understanding the dynamics of the intestinal microbiota, along with the identification of the viruses contributing to pathological effects, are essential prerequisites for a thorough elucidation of the virome's impact on health and disease (Weiss & Hennet, 2017). Research studies have demonstrated that many viral families are likely active in the development of inflammatory processes in the intestine. Here, knowledge of the viral specimens, their interdependence with third microbial community members, the host, and their stress status are of paramount importance for a thorough definition of the mechanisms by which these alterations occur (Matijašić et al., 2020).

Currently, only a few studies tailored for the investigation of the virome fraction are available. Moreover, the scientific community lacks standardized procedures for the characterization of the virome fraction in a comparable and unbiased manner, ranging from wet‐lab standard operative procedures to bioinformatic data processing (Pavia et al., 2023). Much of our knowledge regarding how viral nucleic acid is perceived through recognition receptors in the intestine comes from studies on Norovirus and Rotavirus, considered pathogens rather than commensals since they were major causes of viral gastroenteritis and death in children before the introduction of vaccines. However, Rotaviruses and Noroviruses encode virulence that blocks IFN signaling, likely absent in many commensal virus families that, in turn, might employ this molecular armory in response to unfavorable conditions, leading to inflammation (Iliev & Cadwell, 2021).

Many enteric viruses that infect eukaryotic cells require the support of the bacterial microbial community for efficient infection and viral transmission. The primary mechanisms by which viruses attack host cells include the direct binding of virions through commensal bacteria, enhancing their stability and facilitating attachment to target host cells. Additionally, bacterial flora can indirectly promote infection of eukaryotic cells by acting on the mucosal immune system (Kuss et al., 2011). In contrast to these mechanisms where bacteria promote viral infection, members of the gut microbiota are also involved in orchestrating the host response to viruses. Studies in mice spontaneously resistant to rotavirus infection revealed that segmented filamentous bacteria promote the proliferation and shedding of epithelial cells to block virus infectivity (Shi et al., 2019). This demonstrates that the presence of a single bacterial species can influence the course of enteric viral infection and underscores the importance of joint profiling (bacterial and viral fractions) for a more realistic representation of the microbiota under homeostatic and dysbiotic conditions (Iliev & Cadwell, 2021).

Regarding stress conditions, the studies above provide clear examples of the impact it has on the structural and functional composition of the intestinal microbiota. Nevertheless, it remains to be clarified to what extent this alteration involves the virome, as well as the downstream effects at the systemic level, linked to the various kinds of stressors. Current research highlighted that stress and virome are responsible for effects at both the intestinal and immune levels through a variety of physiological routes. These lead to further effects with strong implications on infectious disease susceptibility and/or the emergence of noninfective systemic disorders.

## 7 CONCLUSION

Although considered a minor fraction of the microbial community inhabiting the gut environment, the elucidation of the intricate network of interactions between stress, the virome, and the host enables a comprehensive definition of the microbiological fingerprint of each subject. This could pave the way for the development of new diagnostic strategies. Furthermore, this knowledge enables the effective modulation of the intestinal microbial community for therapeutic and/or prophylactic purposes through a personalized medicine approach. A clear understanding of the mechanisms of immunosuppression due to chronic and/or acute stress, its influence on microbial dysbiosis, and the onset of intestinal pathologies, whether of infectious or noninfectious origin, could lead to the formulation of probiotics aimed at containing or delaying the onset of the pathological condition.

To date, several questions remain unanswered, ranging from a clear definition of the virome composition across various subjects' conditions and statuses, the unbiased assessment of the interfering variables, the elucidation of the mechanism undertaken in the gut virome alteration, and the relationships the various microbial specimens establish with the superorganism. From these perspectives, new research lines are hoped for to design patient‐tailored interventions, accounting for the state of immune efficiency, neuropsychological status, and microbial composition specific to each subject.

## AUTHOR CONTRIBUTIONS

**Francesca Talarico**: Conceptualization (equal); writing—original draft (lead); formal analysis (lead); writing—review and editing (lead). **Bruno Tilocca**: Conceptualization (lead); writing—original draft (equal); formal analysis (lead); writing—review and editing (lead). **Rocco Spagnuolo**: Conceptualization (supporting); writing—original draft (supporting); formal analysis (supporting); writing—review and editing (equal). **Ludovico Abenavoli**: Conceptualization (equal); writing—original draft (supporting); formal analysis (supporting); writing—review and editing (supporting). **Francesco Luzza**: Conceptualization (equal); writing—original draft (equal); formal analysis (equal); writing—review and editing (equal). **Paola Roncada**: Conceptualization (lead); writing—original draft (equal); formal analysis (equal); writing—review and editing (equal).

## CONFLICT OF INTEREST STATEMENT

None declared.

## ETHICS STATEMENT

None required.
